# Oct4-Induced Reprogramming Is Required for Adult Brain Neural Stem Cell Differentiation into Midbrain Dopaminergic Neurons

**DOI:** 10.1371/journal.pone.0019926

**Published:** 2011-05-31

**Authors:** Michela Deleidi, Oliver Cooper, Gunnar Hargus, Adam Levy, Ole Isacson

**Affiliations:** 1 Center for Neuroregeneration Research, Harvard Medical School/McLean Hospital, Belmont, Massachusetts, United States of America; 2 Udall Parkinson's Disease Research Center of Excellence, Harvard Medical School, Boston, Massachusetts, United States of America; University of Nebraska Medical Center, United States of America

## Abstract

Neural stem cells (NSCs) lose their competency to generate region-specific neuronal populations at an early stage during embryonic brain development. Here we investigated whether epigenetic modifications can reverse the regional restriction of mouse adult brain subventricular zone (SVZ) NSCs. Using a variety of chemicals that interfere with DNA methylation and histone acetylation, we showed that such epigenetic modifications increased neuronal differentiation but did not enable specific regional patterning, such as midbrain dopaminergic (DA) neuron generation. Only after Oct-4 overexpression did adult NSCs acquire a pluripotent state that allowed differentiation into midbrain DA neurons. DA neurons derived from Oct4-reprogrammed NSCs improved behavioural motor deficits in a rat model of Parkinson's disease (PD) upon intrastriatal transplantation. Here we report for the first time the successful differentiation of SVZ adult NSCs into functional region-specific midbrain DA neurons, by means of Oct-4 induced pluripotency.

## Introduction

One of the fundamental questions in the field of regenerative neuroscience is whether adult forebrain subventricular zone (SVZ) neural stem cells (NSCs) can efficiently generate neuronal phenotypes other than their native inhibitory olfactory bulb (OB) interneuron populations. Adult SVZ NSCs are primarily fated to generate non-dopaminergic (DA) gamma-amino butyric acid (GABA)-ergic olfactory bulb (OB) interneurons [1], [2], [3], [4]. This represents an obstacle to the development of successful therapeutic strategies for neurodegenerative diseases, since region-specific phenotypes are warranted for the generation of clinically relevant neurons by mobilization of endogenous neural precursor cells (NPCs) after degeneration or lesion.

With respect of cell therapy for Parkinson's disease (PD), several pieces of evidence now demonstrate the importance of the midbrain DA neuronal subtype as a determinant of the functional impact of cell-based strategies in animal models of PD [5], [6]. The critical challenge is to generate neuronal populations with the phenotypic and molecular properties of midbrain DA neurons in order to achieve proper striatal reinnervation. However, there is still no evidence of the successful manipulation of adult SVZ NSCs toward a midbrain DA neuronal identity suitable for such clinical regenerative purposes. In vitro, midbrain DA neurons have only been efficiently derived from early fetal ventral midbrain and embryonic stem cells (ESCs) from preimplanted blastocysts of embryos [7]. On the contrary, adult SVZ NSCs are more restricted in their capacity to generate neuronal subtypes with a specific regional identity [1], [2], [8]. In vivo, different strategies have been tested in order to promote the proliferation of endogenous SVZ NPCs, their migration toward the lesioned striatum, and their differentiation into midbrain DA neurons [9], [10], [11], [12]. However, there is no evidence that such strategies promote the generation of functional midbrain DA neurons that integrate into the nigrostriatal DA system [13], [14], [15].

During development, adult SVZ NSCs lose their competency for neuronal region-specific patterning and therefore acquire a restricted temporal and regional specification [16]. Epigenetic modifications such as histone acetylation and DNA methylation play an important role in regulating such fate determination [17]. Importantly, DNA methylation and histone acetylation state closely correlates with NSC multipotency both in vivo and in vitro [18].

Here, we sought to investigate whether chromatin-modifying agents (such as histone deacetylase inhibitors and demethylating agents) can regulate the capacity of adult SVZ NSCs to differentiate into region-specific neuronal subtypes such as midbrain DA neurons. We found that chromatin-modifying agents increase neuronal differentiation of adult SVZ NSCs without altering their capacity to differentiate into region-specific neuronal phenotypes. Only by Oct4-induced reprogramming could adult SVZ NSCs re-acquire the competency to differentiate into multiple neuronal lineages.

## Materials and Methods

### Animals

4–8 week old C57BL/6 mice (Charles River Laboratories) and B6;129-Gt(ROSA)26Sor^tm1(rtTA*M2)Jae^ Col1a1^tm2(tetO-Pou5f1)Jae^/J (rTA/Oct4) mice (The Jackson Laboratory) were used in the study. Female Sprague-Dawley rats with unilateral 6-OHDA lesions were obtained from Taconic. All animal procedures were performed in accordance with the guidelines of the National Institutes of Health and were approved by the Institutional Animal Care and Use Committee (IACUC) at McLean Hospital, Harvard Medical School (Mclean IACUC Protocol #09-3/2-6 approved on 09/16/10).

### Primary neural stem cell cultures and differentiation

NSC cultures were established from the lateral ventricular walls of 4- to 8-week-old female mice, as described [19]. Briefly, the SVZ was isolated [20] and digested in 0.1% trypsin-EDTA. Cells were plated at a density of 20 cells/µl in growth medium, consisting of mouse NeuroCult NSC basal medium, mouse NeuroCult NSC proliferation supplements, 2 µg/ml heparin, penicillin [100 U/ml]/streptomycin [100 U/ml], EGF (20 ng/ml) and bFGF (10 ng/ml) (all from StemCell Technologies). To analyse cell proliferation, wild-type (n = 3) or rTA/Oct4 (n = 3) primary NSCs were plated at 8000 cells/cm^2^ in neurosphere growth medium. After 3–4 days, neurospheres were harvested, mechanically dissociated, counted and re-plated under the same culture conditions. For in vitro neurosphere formation assay, NSCs from wild-type (n = 5) and rTA/Oct4 mice (n = 5) were plated in 24-well polyornithine coated plates at a density of 8000 cells/cm^2^ in growth medium with or without doxycycline (DOX) (2 µg/mL; Sigma). Number of neurospheres (diameter≥100 µm) was counted 7 days after plating [21].

To induce differentiation, cells were manually dissociated and plated on glass coverslips coated with 10 µg/mL poly-L-ornithine and 1 µg/mL laminin at density of ≈5∧10^5^ cells/cm^2^. Cells were first expanded and patterned for 4 days after plating in N2 medium supplemented with FGF2 (10 ng/ml), Shh (500 ng/ml), and FGF8 (100 ng/ml) (all from R&D Systems). After 4 days, cells were subsequently differentiated in N2 medium containing ascorbic acid (AA) (200 µM; Sigma) for 10–14 days. In some experiments, NSCs were treated with VPA (1 mM, EMD), TSA (100 nM, EMD), AZA (1 µM, Sigma), BIX-01294 (1 µM, Sigma) or 100 nM TSA/500 nM AZA for 72 hours and then replated in differentiation medium containing 1 mM VPA, 100 nM TSA, 1 µM AZA, 1 µM BIX-01294 or 100 nM TSA/500 nM AZA. After an additional 48 hours, chemicals were withdrawn and differentiation was carried out as described above. Untreated NSCs and dimethyl sulfoxide (DMSO)-treated NSCs were used as control. Three independent experiments were run at least in triplicate.

### Bisulfite sequencing

DNA was isolated with a QIAGEN DNeasy kit. Purified genomic DNA was denatured and converted with an Epitect kit QIAGEN. 500 ng of DNA was then bisulfite converted and purified. Bisulfite sequencing was carried out by Genpathway, Inc. Bisulfite conversion specific primers were designed using MethPrimer. PCR products were cloned into the TOPO-TA vector (Invitrogen), transformed into bacteria and plated on agarose plates. Colonies were picked, inserts were PCR amplified using M13 forward and reverse primers and products were visualized on a 1% agarose gel. Amplified PCR products were sequenced using M13 reverse primers. The following primers were used: bisulfite PCR-forward (GTAATGGTTGTTTTGTTTTGGTTTT), bisulfite PCR-reverse (CCACCCTCTAACCTTAACCTCTAAC).

### Generation of NSC-derived iPSCs

NSCs established from 4 week-old rTA-Oct4 transgenic mice were seeded at a density of 5∧10^5^ cells per 6-well plate in growth medium with DOX (1–2 µg/ml). Forty-eight hours later, cells were further subcultured on irradiated MEFs (GlobalStem) in ESC medium (DMEM supplemented with 15% FBS, nonessential amino acids, L-glutamine, penicillin/streptomycin, β-mercaptoethanol, and 1000 U/ml LIF) with DOX (2 µg/ml). In some conditions, VPA (0.5 mM) was added to ESC medium for 7–10 days. ES-like colonies were mechanically isolated, and individual cells were dissociated and subsequently replated onto MEFs.

### Quantitative Real-Time PCR

Total RNA was extracted with an RNeasy kit (QIAGEN) as described [22]. The expression level of each gene was normalized to endogenous β-actin. Fold-change in gene expression was calculated using 2^−ΔΔCT^ method [23]. All the results are from three technical replicates of three independent experiments. Primer sequences are available on request.

### Western-Blot

Cells were lysed in NE-PER Nuclear and Cytoplasmic Extraction Kit (Pierce) according to the manufacturer's protocol. Protein extract (20 µg) was run on a 4%–15% gradient gel (Bio-Rad). The following primary antibodies were used: Oct-4 (1∶200, Santa Cruz, H-134), β-TubIII (1∶1000, Abcam), GFAP (1∶1000, Millipore), GAPDH (1∶5000, Millipore).

### In vitro differentiation of NPC-derived iPSCs

Cells were harvested by trypsinization and transferred to bacterial culture dishes in ESC medium without LIF. After 3 days, aggregated cells were plated onto gelatin-coated tissue culture dishes and incubated for another 7 days. Differentiated EBs were fixed with 4% paraformaldehyde.

### In vitro differentiation of NSC-derived iPSCs into DA neurons

NPC-derived iPSCs were differentiated into DA neurons using a previously described protocol [7], with some modifications. iPSCs (stage 1) were dissociated and plated in bacterial dishes. EBs were allowed 3 days to form in DMEM containing defined 10% FBS, L-glutamine (2 mM), 1× NEAA, HEPES (10 mM), β-mercaptoethanol (1 mM), penicillin (100 U/ml)/streptomycin (100 g/ml) (stage 2). EBs were allowed 1 day to attach to tissue culture dishes, and neuronal precursors were then selected by incubation in DMEM F-12 medium containing apo-transferrin (50 g/ml), insulin (5 g/ml), sodium selenite (30 nM), fibronectin (250 ng/ml), penicillin (100 U/ml)/streptomycin (100 g/ml) for 9–10 days (stage 3). Cells were subsequently dissociated by 0.05% trypsin, and neuronal precursors were expanded and patterned for 4 days after plating onto polyornithine/laminin-coated plates at a density of 75,000 cells/cm^2^ in N2 medium supplemented with laminin (1 mg/ml), FGF-2 (10 ng/ml), Shh (500 ng/mL) and murine FGF8b (100 ng/mL) (stage 4). The cells were subsequently differentiated in N2 medium containing AA (200 µM) for 10–14 days (stage 5).

### Flow Cytometry and Cell Sorting

Cell sorting was performed as previously described [24]. Briefly, cells were harvested at stage 5 (day 7–10), and stained with primary mouse IgM anti-SSEA-1 antibody (0.4 µg/ml; DSHB) for 20 minutes. Cells were then washed, incubated with Alexa Fluor 488 fluorescent secondary antibody. FACS sorting was performed using a FACSAria cell sorter and FACSDiva software (BD Biosciences). A 100 µm nozzle, sheath pressure of 20–25 psi, and an acquisition rate of 1,000–2,000 events per second were used as the standard conditions. Isotype-matched control antibodies were used to set the gate. Annexin V Apoptosis Detection Kit (BD Pharmingen) was used according to the manufacturer's protocol.

### Transplantation into 6-Hydroxydopamine-Lesioned Rats and Analysis of Drug-Induced Rotational Behaviour

NSC-derived iPSCs, sorted for SSEA1 negative expression, were resuspended in HBSS containing 1 µg/ml GDNF (Sigma-Aldrich) at a density of 5∧10^5^ cells/µL. 6-OHDA lesioned rats were grafted into the lesioned striatum with 4 µl of cell suspension as 2 deposits of 100,000 cells using the following coordinates from bregma: site 1: AP+0.4; ML -3; DV -5.0; site 2: AP -0.5; ML -3.6; DV -5.0. Cells were engrafted at a rate of 0.3 µl per minute. Immunosuppression, anesthesia, transplantation, and analgesia were performed as previously described [25]. Rotational asymmetry of 6-OHDA-lesioned rats was analysed after i.p. injection of amphetamine (4 mg/kg; 90 min) or s.c. injection of apomorphine (0.1 mg/kg; 40 min) two weeks before transplantation and at 4, 6 and 8 weeks after transplantation. Functionally lesioned rats were randomly assigned to 2 groups, one control group (n = 6) and one group for the transplantation of differentiated NSC-iPSCs (n = 5). The animals were sacrificed 8 weeks post-transplantation. Brains were removed, postfixed in 4% paraformaldehyde, equilibrated in 30% sucrose, and sectioned on a freezing microtome in 40-µm coronal slices.

### Immunofluorescent staining and Stereological Procedures

Immunofluorescent staining was performed as previously described [24]. The following primary antibodies were used: sheep/rabbit anti-TH (1∶1,000; Pel-Freez Biologicals), rabbit anti-Pitx3 (1∶250, Zymed), mouse anti-En1 (1∶50, DSHB), goat anti-Foxa2 (1∶100, Santa Cruz), rabbit/chicken anti β-TubIII (Tuj1, Covance), rabbit anti-GFAP (1∶1000, Dako), mouse anti-SSEA-1 (0.4 µg/ml; DSHB), mouse anti Oct4 (1∶100, Santa Cruz Biotechnology), rabbit anti Nanog (1∶100, Bethyl), mouse anti NeuN (1∶1000, Chemicon), mouse anti SSEA1 (1∶50, Chemicon), rabbit anti GAD67 (1∶5000, Sigma), rabbit anti GABA (1∶5000, Sigma), mouse anti AFP (1;100, R&D Systems), goat anti brachyury (1∶100, Santa Cruz Biotechnology). Appropriate fluorescence-labeled secondary antibodies (AlexaFluor; Invitrogen) were used and nuclei were stained with Hoechst 33342 (5 µg/mL; Sigma-Aldrich). For light microscopy, biotinylated secondary antibodies (1∶300, Vector Laboratories) were applied to detect anti-TH antibody, followed by incubation in streptavidin-biotin complex (Vectastain ABC Kit Elite; Vector Laboratories) for 1 h and visualized by incubation in 3,3′-diaminobenzidine (DAB; Vector Laboratories). Alkaline phosphatase (AP) staining was performed using Alkaline Phosphatase.

Detection Kit (Chemicon), according to the manufacturer's instructions. Confocal analysis was performed using a Zeiss LSM510/Meta Station (Thornwood). Stereology was performed using Stereo Investigator image-capture equipment and software (MicroBright-Field,) and a Zeiss Axioplan I fluorescent microscope. Three coverslips were counted for each immunostaining.

### Statistical analysis

For statistical analyses, we used a standard software package (GraphPad Prism version 4.00). Data are expressed as mean±SEM. Asterisks identify experimental groups that were significantly different from control groups by t-test, one-way ANOVA, or two-way ANOVA with a Bonferroni correction for multiple comparisons (p value, 0.05), where applicable.

## Results

### Chromatin modifying agents do not alter the differentiation potential of mouse adult SVZ NSCs

**Figure 1:**
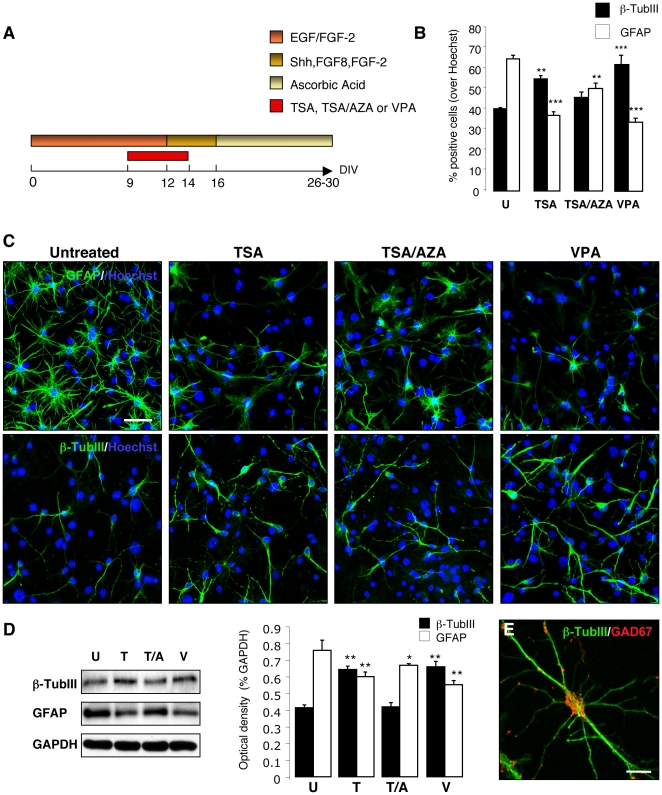
Chromatin modifying agents increase neuronal differentiation of adult mouse SVZ NSCs. (**A**) Schematic representation of the experimental design: adult SVZ NSCs were treated with TSA, TSA/AZA or VPA for 5 days and differentiated using a modified protocol developed for the differentiation of mouse ESCs to midbrain DA neurons. (**B**) Quantification of β-TubIII^+^ neurons and GFAP^+^ astrocytes in untreated, TSA-, TSA/AZA- or VPA-treated cultures. Treatment with TSA and VPA increased the number of β-TubIII^+^ neurons, whereas TSA, TSA/AZA and VPA decreased the number of GFAP^+^ astrocytes. Error bars indicate SEM. Three independent experiments were performed in triplicate (** p≤0.01, *** p≤0.001; One-way ANOVA). (**C**) Immunofluorescence staining for neuron-specific class III β-Tubulin (TUJ1) and the glial-specific marker glial fibrillary acidic protein (GFAP) of untreated, TSA-, TSA/AZA-, and VPA-treated cultures. Nuclei were counterstained with Hoechst. (**D**) Western blot analysis of differentiated NSC extracts, untreated or differentiated in the presence of TSA, TSA/AZA or VPA. Two independent experiments were performed in triplicate. Error bars indicate SEM. (*p≤0.05, ** p≤0.01; One-way ANOVA). Optical densities of the individual bands were quantified using NIH ImageJ and normalized by the averaged value of GAPDH. (**E**) Immunofluorescence staining for β-TubIII (green) and GAD67 (red) of differentiated adult SVZ NSCs treated with VPA. The majority of β-TubIII^+^ neurons co-expressed GAD67. Scale bars: 20 µm (C); 10 µm (E). U, untreated; T, TSA; T/A, TSA/AZA; V, VPA.

We first investigated whether epigenetic modifications induced by chromatin modifying agents can increase adult SVZ NSC responsiveness to the patterning signals that regulate midbrain DA neuron specification such as sonic hedgehog (Shh) and fibroblast growth factor 8 (FGF8). We tested the effects of a variety of chemicals that have been shown to enhance cell dedifferentiation by promoting an ESC-like state: 5-aza-cytidine (AZA), a DNA methyltransferase (DNMT) inhibitor; BIX-01294, a G9a histone methyltransferase inhibitor; valproic acid (VPA) and trichostatin A (TSA), histone deacetylase inhibitors (HDACi) (Table 1) [26]. The non-toxic concentration of each chemical was determined by exposing adult SVZ NSCs to a range of concentrations and determining cell toxicity by Annexin V/Propidium iodide flow-cytometry analysis at 24 and 48 hours after treatment (data not shown). In order to test whether such chromatin modifying agents increase NSC competency to region-specific neuronal differentiation, NSCs were isolated from the SVZ of adult C57/BL6 mice and grown as neurospheres in the presence of epidermal growth factor (EGF) and fibroblast growth factor 2 (FGF-2). In order to limit the exposure to EGF and FGF-2 that can deregulate the spatial identity and differentiation potential of neural precursors [27], only primary neurospheres were used in these experiments. NSCs were treated with 1 µM AZA, 1 µM BIX-01294, 1 mM VPA, 100 nM TSA, or 500 nM AZA/100 nM TSA (Table 1) for 72 hours. NSCs were then differentiated upon EGF withdrawal, using a modified protocol developed for the differentiation of mouse ESCs to midbrain DA neurons [7], [24] (Fig. 1A). In this protocol, the signaling molecules Shh, FGF-2 and FGF8 induce ventral midbrain DA neuron patterning of neuronal precursors. Final differentiation to mature DA neurons is induced by ascorbic acid (AA) in the absence of growth factors. Differentiation medium was supplemented with 1 µM AZA, 1 µM BIX-01294, 1 mM VPA, 100 nM TSA, or 500 nM AZA/100 nM TSA for an additional 48 hours (Fig. 1A). AZA alone and BIX-01294 at effective concentrations were found to be too toxic when added during differentiation, thus were excluded from further studies. Treatment with HDACi (TSA,VPA) induced a significant increase of β-TubIII^+^ neurons as revealed by immunohistochemistry at day 30 (40.5±0.5%, 55.25±1.6%, 62.5±4% in untreated, TSA and VPA treated cells; p≤0.01 and p≤0.001 compared to untreated respectively) and western blot analysis (Fig. 1B–D). Treatment with TSA, TSA/AZA and VPA decreased the number of GFAP^+^ astrocytes in differentiated cultures at day 30 (65±1.9%, 37.25±1.8%, 48.34±1.5%, 34±1.9% in untreated, TSA, TSA/AZA, and VPA treated cells; p≤0.001, p≤0.01 and p≤0.001 compared to untreated cells) (Fig. 1B–C). Western blot analysis confirmed the decrease of astrocytic differentiation after treatment with TSA, TSA/AZA and VPA (Figure 1D). We examined the expression of tyrosine hydroxylase (TH), the rate-limiting enzyme of DA synthesis, and other midbrain DA neuronal markers [engrailed-1 (En1), paired-like homeodomain transcription factor 3 (Pitx3), dopamine transporter (DAT), and nuclear receptor related 1 (Nurr1)] by immunocytochemistry and RT-PCR at day 16, 26 and 30. Some TH^+^ neurons were transiently detected at day 16 in HDACi treated cultures (data not shown). However, after the completion of the differentiation protocol (day 30), differentiated neurons did not express any midbrain DA marker. We found that differentiated neurons almost exclusively expressed GAD67, a marker for GABAergic interneurons (Fig. 1E).

**Table 1:** List of reagents used in the study.

Chemical	Function	Concentration
**AZA**	DNMT inhibitor	0.5–1 µm
**BIX-01294**	G9a histone methyltransferase inhibitor	1 µm
**VPA**	HDAC inhibitor	1 mM
**TSA**	HDAC inhibitor	100 nM

### Epigenetic modifiers increase the expression of pluripotency-associated genes and partial demethylation of *Oct4* promoter

**Figure 2:**
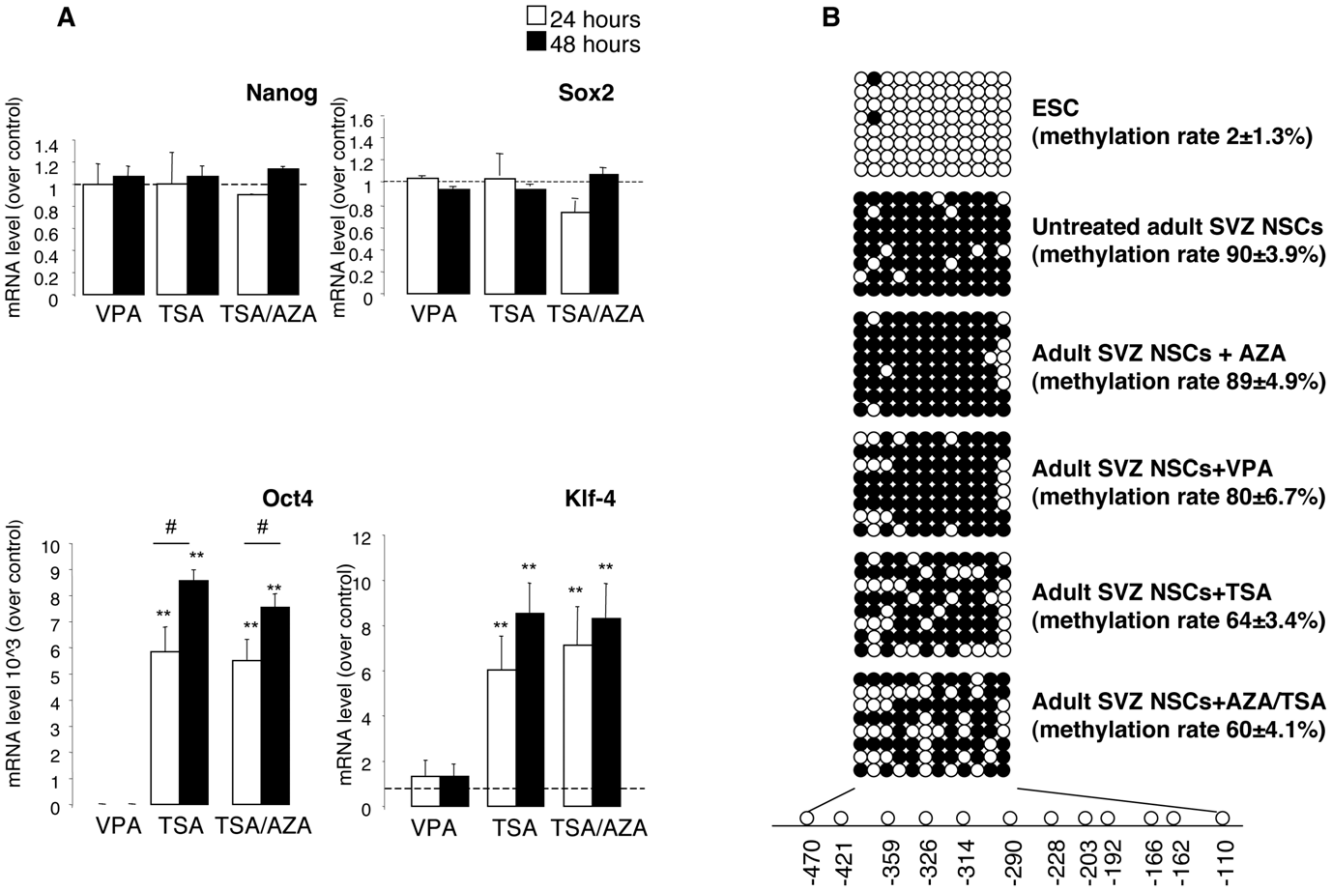
Chromatin modifying agents induce the expression of pluripotency-associated genes and promote partial demethylation of *Oct4* promoter in adult SVZ NSCs. (**A**) SVZ adult NSCs were treated with VPA, TSA, or TSA/AZA and mRNA level of Nanog, Sox2, Oct4 and Klf4 was analysed by qRT-PCR. Values were normalized to the level of β-actin and expressed as relative increase over vehicle-treated cultures. Error bars indicate SEM. Three independent experiments were performed in triplicate (** p≤0.01; # p≤0.05; One-way ANOVA). (**B**) The DNA methylation profile of 12 CpG sites located in the *Oct4* proximal promoter from −469 to the ATG start codon in mouse ESCs, untreated SVZ NSCs and NSCs treated with AZA, TSA, VPA or AZA/TSA is shown. The methylated and non-methylated CpG positions are presented as black and white circles, respectively. Ratios indicate average of methylated to unmethylated sites at the 12 CpG sites±SEM.

We then analysed the expression of pluripotency-associated genes such as Nanog, Sox2, Oct4, and Klf4 in adult SVZ NSCs at 24 and 48 hours after treatment with VPA, TSA or TSA/AZA. We found that TSA alone or TSA/AZA significantly induced the expression of Oct4 and Klf4 at 24 and 48 hours (Fig. 2A). We investigated the DNA methylation profile of *Oct4* promoter. Oct-4 is the master regulator of stem cell pluripotency and differentiation [28] and the methylation of its promoter drives the conversion from primitive NSCs (pNSCs) to definitive NSCs (dNSCs) [29], thus limiting their competency to undergo region-specific neuronal differentiation. The methylation status of 12 CpG sites in the *Oct4* promoter region (between 470 to the ATG start codon) was assessed by bisulfite sequencing in mouse ESCs, untreated adult SVZ NSCs and adult SVZ NSCs treated with AZA, VPA, TSA or AZA/TSA for 48 hours (Fig. 2B). CpG sites within *Oct4* promoter were mostly unmethylated in mouse ESCs (methylation rate: 2.075±1.3%). Conversely, untreated NSCs and AZA-treated NSCs were highly methylated (methylation rate: 90±3.9% and 89.9±4.9%, respectively). Upon treatment with VPA, TSA or AZA/TSA we found more unmethylated CpG sites (methylation rate: 80±6.7%, 64±3.4%, 60±4.1%, respectively) (Fig. 2B). These data indicate that the chromatin-modifying agents (TSA, VPA and TSA/AZA) induced de novo expression of pluripotency genes and partial demethylation of *Oct4* promoter in adult SVZ NSCs. However, these changes were not sufficient to reverse NSC fate restriction and induce the competency to patterning by midbrain developmental cues.

### The germline factor Oct-4 increases adult SVZ NSC proliferation and self-renewal

**Figure 3:**
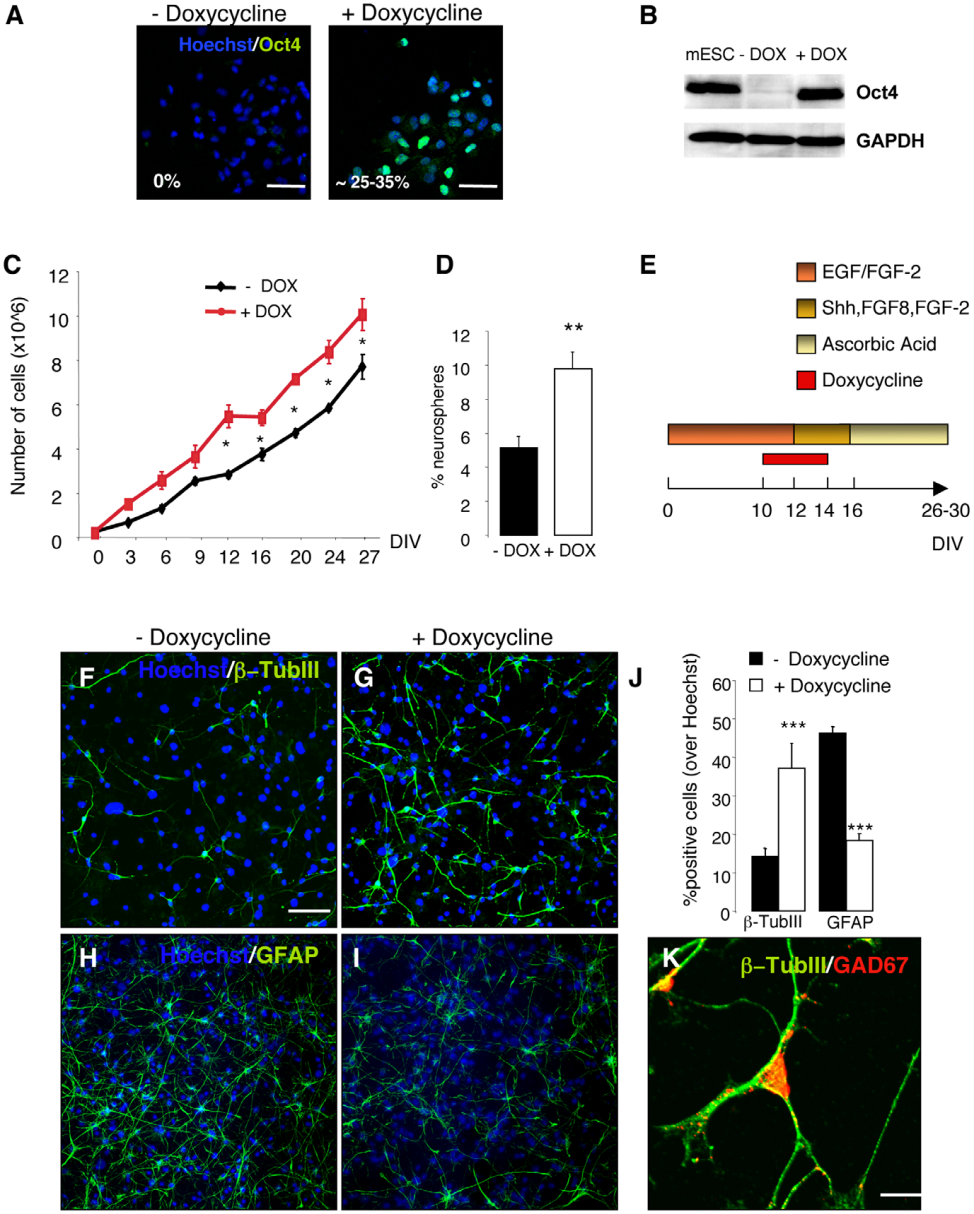
Short-term overexpression of Oct4 increases proliferation, self-renewal and neuronal differentiation of adult SVZ NSCs. (**A**) Adult SVZ NSCs were isolated from doxycycline (DOX)-inducible Oct4 transgenic mice (rTA-Oct4) and grown as neurospheres in growth medium with EGF/FGF-2. Oct4 was induced by 2 µg/mL DOX. Fluorescence images of rTA-Oct4 NSCs cultured for 48 hours, with or without DOX, and stained with an antibody to Oct4. Percentages of Oct4^+^ cells are indicated. (**B**) DOX-dependent induction of Oct-4 protein in adult SVZ NSCs from rTA-Oct4 transgenic mice determined by Western blot analysis. Mouse embryonic stem cells (mESC) were used as control. GAPDH was used as loading control. (**C**) Growth curves of DOX-induced and -uninduced adult SVZ NSCs. Time points represent average values from triplicate measurements and their standard deviations (* p≤0.05, Two-way ANOVA with post hoc analysis by Bonferroni test). (**D**) Clonal analysis of SVZ adult NSCs after Oct4 induction. NSCs were grown as neurospheres and treated with DOX or left untreated. After dissociation, single cells were replated and the total number of neurospheres with a diameter >100 µm was assessed and expressed as % over plated cells (** p≤0.01, t-test). Error bars indicate SEM. (**E**) Schematic representation of the experimental design: SVZ adult NSCs were isolated from rTA-Oct4 transgenic mice and grown as neurospheres in growth medium with EGF/FGF-2, with or without DOX. Forty-eight hours after Oct4 induction, neurospheres were dissociated and replated in differentiation medium with Shh, FGF8, FGF-2, with or without DOX. DOX was withdrawn after 48 hours. Cells were finally differentiated with ascorbic acid (AA). (**F–I**) Immunofluorescence staining for β-TubIII (green, F–G) and GFAP (green, H–I) of adult SVZ NSCs differentiated with or without Oct4 induction. Nuclei were counterstained with Hoechst. (**J**) Quantification of β-TubIII^+^ neurons and GFAP^+^ astrocytes in untreated and DOX-treated neuronal cultures. Error bars indicate SEM. Three independent experiments were performed in triplicate. (*** p≤0.001; t-test). (**K**) Immunofluorescence staining for β-TubIII (green) and GAD67 (red) of differentiated adult SVZ NSCs upon Oct4 induction. The majority of β-TubIII^+^ neurons co-expressed GAD67. Scale bars: 50 µm (F–I); 10 µm (K).

We then examined whether overexpression of Oct-4 can alter the region-specific differentiation potential of adult SVZ NSCs. We established neurosphere cultures from the lateral ventricular walls of rTA/Oct-4 mice [30]. These mice express rtTA-M2, an optimized form of reverse tetracycline-controlled transactivator (rtTA) protein directed to multiple tissues by the Gt(ROSA)26Sor promoter. Following doxycycline (DOX) administration, high Oct-4 expression is induced in several tissues [30]. In vivo, Oct-4 expression was not originally detected in the brain probably due to the low penetration of DOX through the blood brain barrier (BBB) [30]. Therefore, we first tested Oct-4 expression in adult SVZ NSC cultures derived from rTA/Oct-4 mice. We found that 25-35% of cells expressed Oct-4 as revealed by immunocytochemistry and western-blot analysis 48 hours after DOX induction (Fig. 3A–B). NSCs that were not exposed to DOX did not show any detectable Oct-4 expression (Fig. 3A–B). We then determined the effect of Oct-4 induction on adult SVZ NSC proliferation and self-renewal. rTA/Oct-4 NSCs were grown as neurospheres in the presence of EGF and FGF-2, with or without DOX, and propagated for up to 8 passages. Growth curves revealed increased long-term proliferation rate in DOX-treated NSCs (Fig. 3C). NSC self-renewal was assessed by the neurosphere-colony forming assay. We found more neurosphere-forming cells in DOX-treated cultures (Fig. 3D).

We then investigated whether Oct-4 overexpression in adult SVZ NSCs alters their differentiation potential. Oct-4 was induced by DOX and 72 hours later, NSCs were differentiated upon EGF withdrawal in the presence of Shh, FGF-2 and FGF8 for 4 days. DOX was added to the differentiation medium for an additional 48 hours. Cells were then terminally differentiated with AA for 10–14 days (Fig. 3E). Interestingly, we found that Oct-4 overexpression generated more β-TubIII^+^ neurons (Fig. 3F–G, J) and fewer GFAP^+^ astrocytes (Fig. 3H–I, J). However, at the end of differentiation (day 30), almost all β-TubIII^+^ neurons co-expressed the GABA-ergic marker GAD67 (Fig. 3K).

### Adult SVZ NSCs are reprogrammed to a pluripotent state by exogenous Oct4

**Figure 4:**
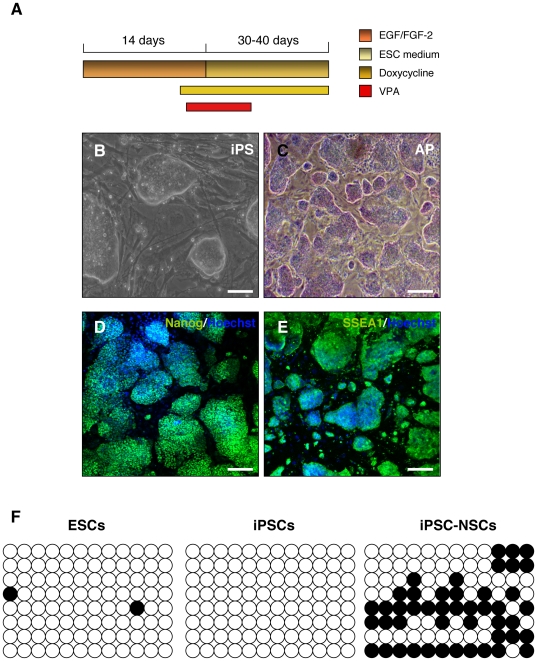
SVZ adult NSCs are reprogrammed to pluripotent stem cells by overexpression of Oct-4. (**A**) Schematic representation of the experimental design: SVZ adult NSCs were isolated from rTA-Oct4 transgenic mice and grown as neurospheres. Oct4 was induced by DOX, and 48 hours later, NSCs were plated on mouse feeder cells (MEF) in ESC medium with 0.5 mM VPA. ESC-like colonies were picked 30–40 days after DOX induction. (**B**) Phase contrast image showing ESC-like morphology of NSC-derived iPSCs (clone mm3) on MEF. (**C–E**) Colonies expressed alkaline phosphatase (AP) and stained positive for Nanog and SSEA-1. (**F**) Methylation analysis of the *Oct4* promoter region. White circles indicate unmethylated and black circles methylated CpGs in the *Oct4* promoter of mouse ESCs, reprogrammed NSCs and NSCs derived from iPSCs (stage 3, day 11). Scale bars: 100 µm.

**Figure 5:**
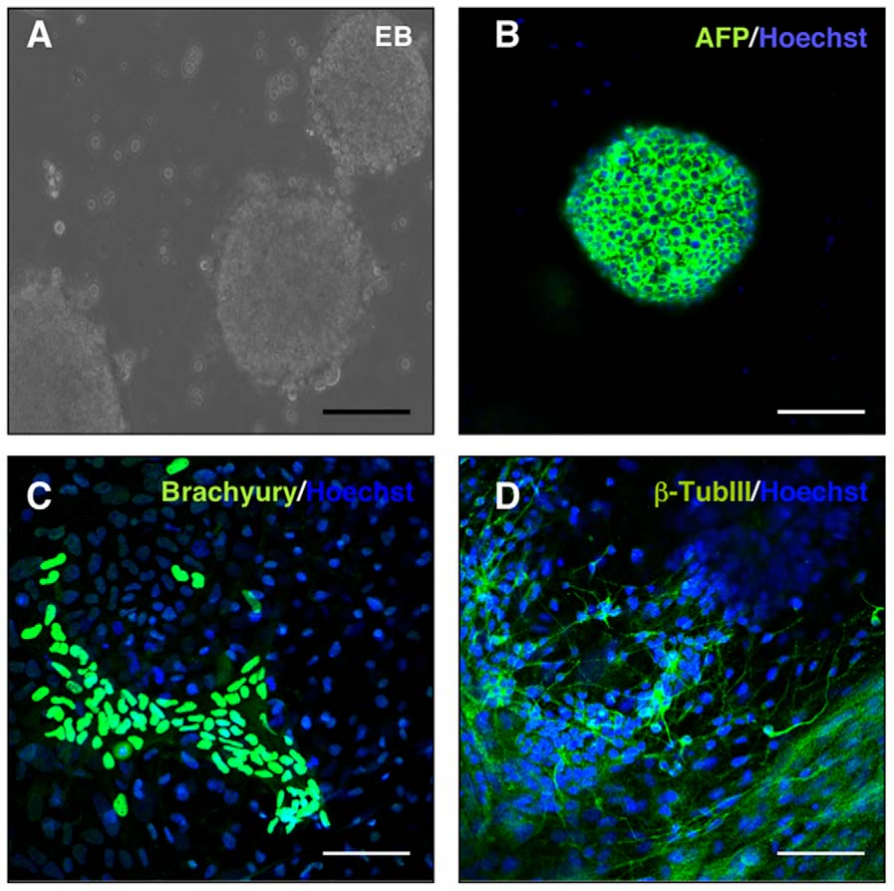
NPC-derived iPSCs can be differentiated into three germ layers in vitro. NSC-derived iPSCs were differentiated into three germ layers by embryoid body (EB) differentiation. (**A**) In vitro EB formation. (**B–D**) EBs expressed markers of the three germ layers including α-fetoprotein (AFP) (endoderm), brachyury (mesoderm), and β-TubIII (ectoderm). Scale bars: 25 µm (A–C); 50 µm (D).

We overexpressed Oct-4 in rTA-Oct4 SVZ NSC cultures by DOX and 48 hours later NSCs were plated on mouse embryonic fibroblasts (MEF) in ESC medium. In order to improve the efficiency of reprogramming, ESC medium was supplemented with 0.5 mM VPA for 7–10 days [31] (Fig. 4A). After 5–6 weeks, we were able to select mouse ESC-like colonies [from now on termed as NSCs-derived induced pluripotent stem cells (iPSCs)]. We characterized 2 NSC-derived iPSC clones (clones mm3 and mm4) (Fig. 4B). The estimated reprogramming efficiency of NSCs was 0.001%. We observed no colonies when VPA was omitted. NSC-derived iPSCs expressed alkaline phosphates (AP), Nanog and SSEA-1, and were morphologically indistinguishable from mouse ESCs (Fig. 4 B–E). As expected, the *Oct4* promoter region of NSC-derived iPSCs was found to be hypomethylated (Fig. 4F). Interestingly, the *Oct4* promoter region of NSCs derived from iPSCs was hypomethylated compared to adult SVZ NSCs (Fig. 4F and 2B). These iPSC colonies could be expanded up to 25 passages. We then examined the ability of NSC-derived iPSCs to differentiate into the three germ layers by embryoid body (EB) differentiation. EBs derived from NSC-iPSCs expressed markers of the three germ layers including α-fetoprotein (AFP) (endoderm), brachyury (mesoderm), and β-TubIII (ectoderm) as determined by immunocytochemical analysis (Fig. 5 A–D).

### Reprogrammed adult SVZ NSCs are successfully differentiated into midbrain DA neurons

**Figure 6:**
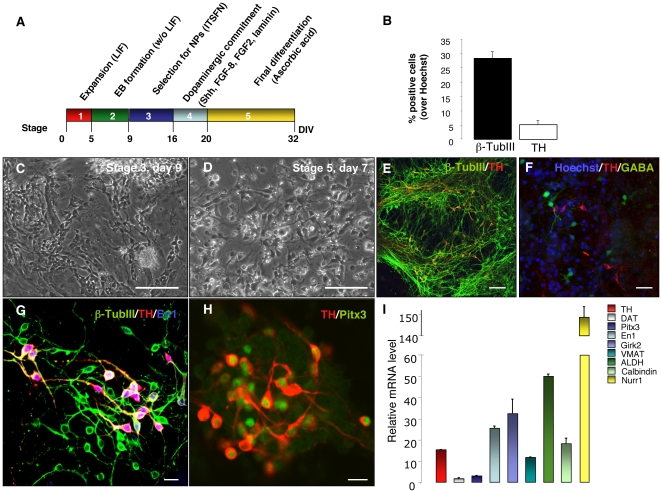
Reprogrammed adult NSCs can be differentiated into midbrain DA neurons. (**A**) NSC-derived iPSCs were differentiated into DA neurons according to the five-stage protocol. Neural differentiation was induced by EB formation in ITSFn medium. Subsequently, the neural precursors were expanded in the presence of the growth factors FGF-2, FGF8, and Shh. Terminal differentiation was induced by growth factor withdrawal in the presence of ascorbic acid. (**B**) Graphs indicate the percentage of cells that stained positive for β-TubIII and TH, relative to nuclear Hoechst staining. Error bars indicate SEM. Three independent experiments were performed in triplicate. (**C**) Brightfield images showing EB-derived FGF2-responsive neural precursor cells. (**D**) Brightfield microphotographs of differentiated NSC-derived iPSCs, 7 days after growth factor withdrawal. (**E**) Immunofluorescence staining of neuronal cultures derived from NSC-derived iPSCs for β-TubIII (green) and TH (red). (**F**) Immunofluorescence staining for TH (red) and GABA (green), a marker typically expressed in olfactory bulb (OB) glomerular interneurons. Nuclei were counterstained with Hoechst. (**G–H**) Confocal images of neuronal cultures stained for β-TubIII (green) (G), TH (red) (G–H), En1 (blue) (G) and Pitx3 (green) (H). (**I**) Quantitative RT-PCR analysis of midbrain transcription factors, DA neurotransmission markers (TH, DAT, Pitx3, En1, Girk2, VMAT, ALDH, Nurr1) and calbindin in differentiated NSC-derived iPSCs. cDNA was isolated from differentiated adult NSC-derived iPSCs, and values were normalized to the level of β-actin. Error bars indicate SEM. Three independent experiments were performed in triplicate. Scale bar: 200 µm (C–D); 50 µm (E–F); 20 µm (G–H). ITSFn, insulin/transferrin/selenium/fibronectin.

To test whether NSC-derived iPSCs were able to generate DA neurons in vitro, we differentiated iPSCs according to the mouse ESC five-stage protocol, with some modifications (Fig. 6A) [7], [24]. We induced neural differentiation by EB formation in ITSFn medium (Fig. 6C). Subsequently, the neural precursors were expanded in the presence of the growth factors Shh, FGF-2 and FGF8. Terminal differentiation was induced by growth factor withdrawal in the presence of AA (Fig. 6D). At day 32, we detected a total of 30% β-TubIII^+^ neurons and a total of 5% TH^+^/β-TubIII^+^ neurons (Fig. 6B, E). To assess neuronal regional specification, we double-labeled TH-positive neurons with antibody against Pitx3 and En1, markers typically expressed in midbrain DA neurons (Fig. 6 G–H). Importantly, the vast majority of TH-positive neurons stained positive for these midbrain markers, suggesting their proper midbrain regional specification in vitro (Fig. 6 G–H). We did not find any colocalization between TH and GABA, a marker typically expressed in OB glomerular interneurons (Fig. 6F). qRT-PCR analysis further confirmed expression levels of midbrain DA markers in differentiated cultures: DAT, PITX3, En1, G-protein-activated inwardly rectifying potassium channel subunit (Girk2), vesicular monoamine transporter (VMAT), aldehyde dehydrogenase 2 (ALDH), Calbindin and Nurr1 (Fig. 6I).

### Midbrain DA neurons from reprogrammed adult SVZ NSCs are successfully grafted and reverse drug-induced behaviour in parkinsonian rats

**Figure 7:**
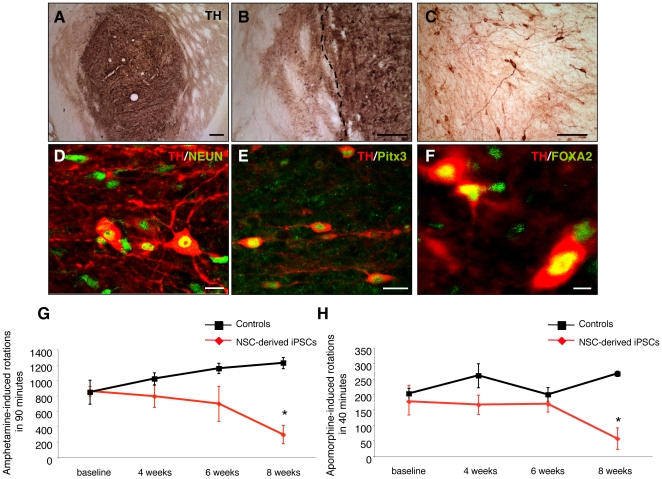
DA neurons from Oct4 reprogrammed adult NSCs integrate into the striatum of 6-OHDA lesioned parkinsonian rats and improve behavioural deficits. (**A**) A low-power overview of an iPSC cell graft 8 weeks after transplantation stained with an antibody against TH (dark brown). (**B–C**) Higher magnification of a NSC-derived iPSC graft showing TH-positive soma and the reinnervation of the surrounding host striatum by donor-derived neurites. The dashed line indicates the edge of the graft. (**D–F**) Confocal analysis of NSC-derived iPSC grafts, 8 weeks post-transplantation, showed that most grafts contained midbrain DA neurons. The grafted TH-positive cells (red) were colabeled with antibodies against NeuN (green) (D), Pitx3 (green) (E) and FOXA2 (green) (F). (**G–H**) DA neurons from NSC-derived iPSCs reversed amphetamine- and apomorphine-induced rotational behaviour upon engraftment into 6-OHDA-lesioned rats. Animals were analysed for amphetamine- and apomorphine-induced rotational behaviour before and 4, 6, and 8 weeks post transplantation. Graphs show mean values±SEM. (* p≤0.05, Two-way ANOVA with post hoc analysis by Bonferroni test). Scale bars: 200 µm (A–B); 100 µm (C); 20 µm (D–E); 10 µm (F).

To further analyse the DA fate potential of NSC-derived iPSCs in vivo, we explored the ability of differentiated neurons to survive, integrate and reinnervate the host striatum of 6-hydroxydopamine (6-OHDA)-lesioned rats, a rodent animal model of PD. Adult NSC-derived iPSCs (clone mm3) were differentiated into DA neurons as described above and transplanted into the striatum of 6-OHDA-lesioned rats. Animals received a striatal graft of 2×10∧5 differentiated cells. In order to avoid tumor formation, SSEA1+ cells were eliminated by cell sorting FACS prior to transplantation (Figure S1). Analysis of amphetamine-induced behaviour showed a significant reduction of ipsilateral rotations at 8 weeks after transplantation (Fig. 7G), indicating significant restoration of the 6-OHDA lesion. We found that rats transplanted with differentiated NSC-derived iPSCs showed a significantly reduced number of apomorphine-induced rotations 8 weeks after engraftment, when compared to the non-transplanted rats (Fig. 7H). Eight weeks after surgery, the animal brains were prepared for morphological analysis. TH staining showed that animals displaying improvement in the behavioural assays had grafts containing large numbers of DA neurons (Fig. 7A–D). Importantly, the transplanted neurons expressed the midbrain DA marker Pitx3 and Foxa2 (Fig. 7E–F). TH-immunoreactive fibres were found to extend into the parenchyma of the host striatum (Fig. 7B,C).

## Discussion

NSCs acquire a regional identity already at the formation of the neural plate during embryogenesis [32], [33]. Therefore, fetal and adult NSCs have passed crucial checkpoints that restrict their developmental capacity to generate region-specific neuronal subtypes.

Epigenetic modifications (i.e. histone code and DNA methylation) play a critical role in regulating adult NSC differentiation and fate determination [34]. Here we investigated whether epigenetic remodeling induced by chromatin-modifying agents can reverse adult SVZ NSC developmental restriction and enable their differentiation into region-specific neuronal subtypes such as midbrain DA neurons. Chromatin remodeling factors can improve the induction of an ESC-like state and have been extensively used to enhance dedifferentiation of somatic cells to the pluripotent state [31]. We showed that chromatin-modifying agents (TSA and TSA in combination with AZA) reactivate specific pluripotency-associated genes such as Oct4 and Klf-4 in adult SVZ NSCs. These agents induced partial demethylation of *Oct4* promoter, thus promoting a dedifferentiation toward a primitive neural stage. We observed an increased neuronal differentiation, paralleled by a decreased number of astrocytes in treated NSC cultures. However, these modifications were not sufficient to reverse the resistance of NSCs to the patterning signals that regulate midbrain DA development, since the majority of differentiated neurons showed a GABAergic phenotype. Previous reports have shown the derivation of iPSCs from NSCs [35], [36]. Specifically, the overexpression of Oct-4 alone induces the reprogramming of NSCs obtained from mouse whole brain and fetal human telencephalon NSCs [37], [38]. In the current work we show for the first time that Oct4 overexpression in association with the chromatin-modifying agent VPA induced adult SVZ NSC reprogramming into iPSCs. Such Oct4-reprogrammed NSCs were then successfully differentiated into midbrain DA neurons. Importantly, we show for the first time that single-factor reprogrammed adult SVZ NSCs generated functional midbrain DA neurons that successfully improved the motor behavioural deficits in a rodent model of PD. Such functional motor behavioural recovery in this model is further significant evidence for the correct subtype of DA neurons generated from NSC-derived iPSCs.

With respect to functional cell therapy for the motor symptoms of PD, it is critical to obtain DA neurons that have the molecular properties of midbrain DA neurons [6]. Both substantia nigra (SN-A9) and ventral tegmental area (VTA-A10) DA neurons contribute to widespread and dense axonal arborization [39], and SN-A9 DA neurons are responsible for appropriate striatal reinnervation and behavioural motor recovery in rodent models of PD [5], [6]. However, no studies have reported successful in vitro differentiation of adult SVZ NSCs into midbrain DA neurons. When grown as neurospheres and differentiated according to the most dedicated differentiation culture protocols, fetal and adult NSCs only generate a small number of TH^+^ neurons [40]. Furthermore, such differentiated TH^+^ neurons do not express markers of midbrain DA neurons [41], [42], which is the required cell type for grafting and appropriate striatal reinnervation in PD. In vivo, mobilization of SVZ endogenous precursor cells has been envisaged as a promising alternative to cell transplantation for the treatment of neurodegenerative diseases including PD [43]. Therefore, different approaches have been tested to promote the differentiation of adult SVZ NSCs to midbrain DA neurons and endogenous NPC proliferation in order to achieve tissue repair and functional recovery in several animal models of neurodegenerative diseases [9], [10], [11], [12], [44]. However, there is no evidence that such strategies promote the generation of functional midbrain DA neurons that integrate into the injured or naïve nigrostriatal DA system [13], [14], [15].

Adult NSCs are resistant to the signaling molecules (Shh, FGF8) that control the development of midbrain and hindbrain [45]. The precise mechanism by which such restriction is controlled and maintained in adult SVZ NSCs is still poorly understood. During mouse development, the earliest NSCs can be isolated starting at embryonic day 5.5 (E5.5) [32]. These pNSCs retain ESC characteristics such as high Oct-4 expression and in vitro LIF-dependence [46]. pNSCs are highly responsive to regionalization cues, allowing the efficient generation of region-specific neuronal subtypes. LIF-dependent NSCs with similar antigenic and functional properties can also be isolated from E5.5–7.5 mouse embryos [47]. Between embryonic day 7.5 (E7.5) and E8.5, germ cell nuclear factor (GNFC)-mediated *Oct4* promoter methylation drives the conversion from pNSCs to dNSCs [29]. In the developing neuroectoderm and NSC cultures, such transition from pNSCs to dNSCs restricts the potential of dNSCs to form non-neural cell types and their capacity to generate region-specific neuron populations.

Interestingly, we found that Oct-4 overexpression, without reprogramming to an ESC state, increased NSC self-renewal and long-term proliferation in the presence of EGF and FGF-2. In addition, we showed that Oct4 overexpression increases neuronal differentiation of NSC cultures in the presence of Shh, FGF-2 and FGF8. However, our data indicate that such short-term Oct-4 overexpression alone does not confer competence for adult SVZ NSC midbrain regionalization. In contrast, long-term overexpression (30–40 days) of Oct-4 in adult SVZ NSCs grown on MEFs in ESC medium with LIF induced complete dedifferentiation to a pluripotent state. Only after Oct-4 induced reprogramming to a pluripotent state were adult SVZ NSCs successfully patterned to midbrain DA neurons.

### Conclusion and summary

In summary, we show that epigenetic modifications are not sufficient to reverse the resistance of SVZ NSCs to the patterning signals that regulate midbrain regional specification. Such modifications do not promote the dedifferentiation of adult SVZ dNSCs toward a primitive neural stage. The current work describes for the first time the reprogramming of adult SVZ NSCs by means of Oct4 overexpression and provides the first evidence that functional midbrain DA neurons can be derived from Oct4-reprogrammed adult SVZ NSCs. These results indicate that the complex and precise regulatory processes responsible for the regional specification of adult NSCs are irreversible, and further de-differentiation steps are required to regain the competency to generate region-specific neuronal phenotypes. These findings have major fundamental scientific and practical implications for regenerative neuroscience.

## Supporting Information

Figure S1 **Elimination of SSEA1+ cells from NSC-derived iPSC neuronal cultures by cell sorting. (A–C)** Neuronal NSC-derived iPSC cultures after sorting based on SSEA1 expression. After sorting, cells were replated onto tissue culture dishes in N2 medium with AA. Three days after sorting, SSEA1^−^ sorted cells displayed mostly neuronal morphology, whereas the SSEA1^+^ sorted cells exhibited an undifferentiated ES cell morphology. **(D–F)** Immunofluorescence images of neuronal cultures 3 days after sorting stained for β-TubIII (green), TH (red) and SSEA1 (blue). Scale bars: 50 µm (A–F).
(TIF)
