# Supplementary figures and images for: Oct4-Induced Reprogramming Is Required for Adult Brain Neural Stem Cell Differentiation into Midbrain Dopaminergic Neurons
Source: PLoS One. 2011 May 31;6(5):e19926. doi: 10.1371/journal.pone.0019926 (PMC3104995; doi:10.1371/journal.pone.0019926)

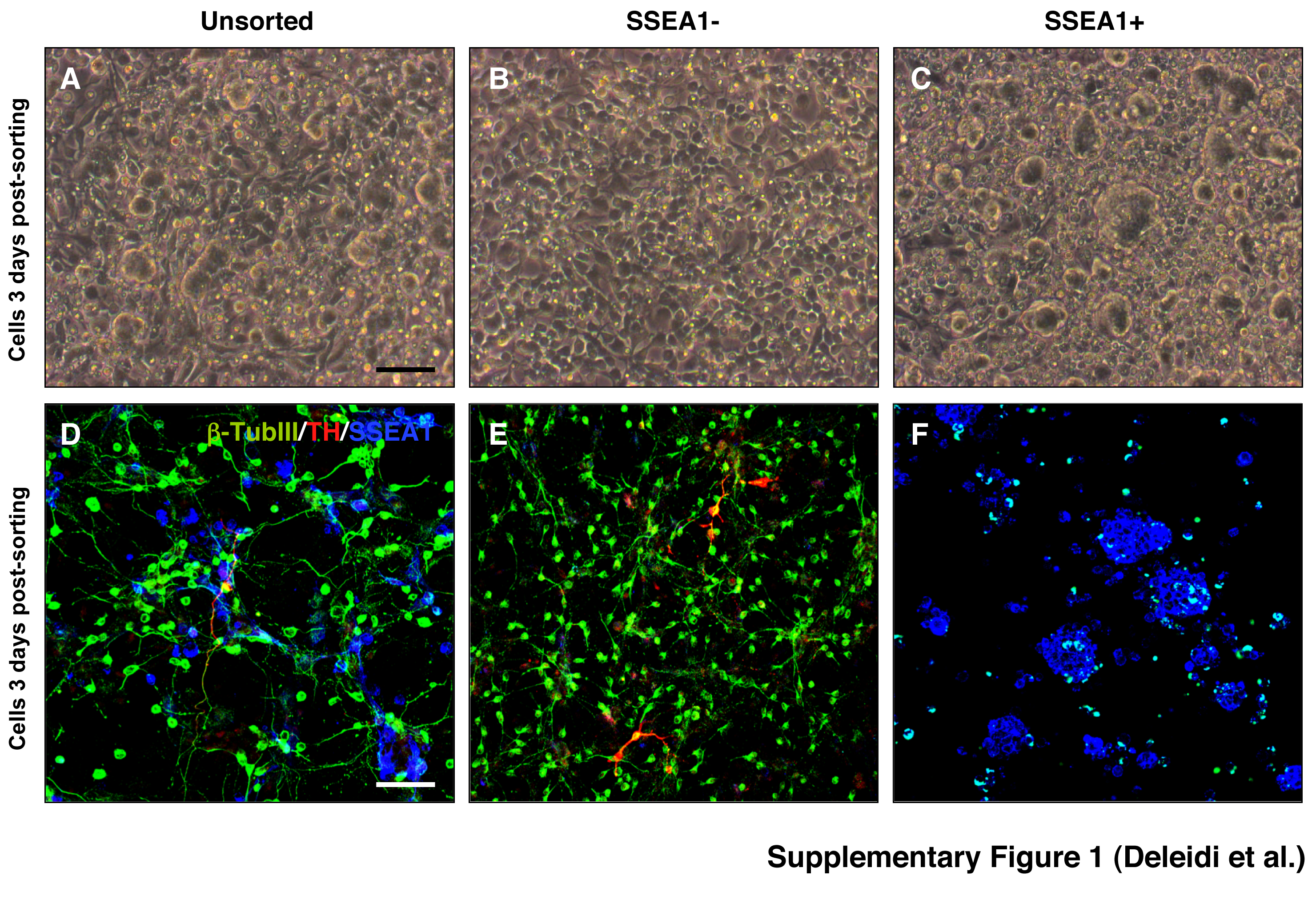

Supplement: Figure S1 — Elimination of SSEA1+ cells from NSC-derived iPSC neuronal cultures by cell sorting. (A–C) Neuronal NSC-derived iPSC cultures after sorting based on SSEA1 expression. After sorting, cells were replated onto tissue culture dishes in N2 medium with AA. Three days after sorting, SSEA1− sorted cells displayed mostly neuronal morphology, whereas the SSEA1+ sorted cells exhibited an undifferentiated ES cell morphology. (D–F) Immunofluorescence images of neuronal cultures 3 days after sorting stained for β-TubIII (green), TH (red) and SSEA1 (blue). Scale bars: 50 µm (A–F). (TIF) [file pone.0019926.s001.tif]
